# Speech Characteristics Across Motor Subtypes of Parkinson's Disease

**DOI:** 10.1111/1460-6984.70081

**Published:** 2025-07-08

**Authors:** Vanessa Brzoskowski dos Santos, Amanda Lara Bressanelli, Fernanda Venzke Zardin, Rui Rothe‐Neves, Maira Rozenfeld Olchik

**Affiliations:** ^1^ Postgraduate Program in Medicine, Medical Sciences Universidade Federal do Rio Grande do Sul Porto Alegre RS Brazil; ^2^ Undergraduate in Speech‐Language Pathology, Universidade do Rio Grande do Sul Porto Alegre RS Brazil; ^3^ Phonetics Lab, Faculdade de Letras Universidade Federal de Minas Gerais Belo Horizonte MG Brazil; ^4^ Neurology Service Hospital de Clínicas de Porto Alegre Porto Alegre RS Brazil; ^5^ Department of Surgery and Orthopedics Universidade Federal do Rio Grande do Sul Porto Alegre RS Brazil

**Keywords:** nonmotor, Parkinson's disease, speech acoustics, speech disorders, tremor

## Abstract

**Background:**

Speech differences may occur between motor subtypes of Parkinson's disease (PD), but the literature remains limited.

**Aims:**

Examine speech characteristics in individuals with PD across the tremor‐dominant, nontremor‐dominant and mixed subtypes comparing to healthy controls.

**Methods and Procedures:**

A total of 115 individuals with PD were included in the study, classified as tremor‐dominant (*n* = 61), nontremor‐dominant (*n* = 39) and mixed (*n* = 15) subtypes according to the Movement Disorder Society – Unified Parkinson's Disease Rating Scale. The control group (CG) consisted of 15 individuals. Speech samples were collected through sustained vowel /a/, diadochokinesis (/pataka/), and monologue and were analysed using both auditory perceptual and acoustic analyses.

**Outcomes and Results:**

In the diadochokinesis and monologue task, the nontremor‐dominant subtype showed shorter production time and, consequently, produced fewer syllables than the CG. The mixed subtype, on the other hand, did not differ from the CG and performed similarly across all tasks. However, the average duration of the syllables in the monologue task significantly differed between the mixed and nontremor‐dominant subtypes.

**Conclusions and Implications:**

There are speech variations among PD motor subtypes. The nontremor‐dominant subtype exhibited poorer speech performance, while the mixed subtype's speech patterns were more similar to those of the CG.

**WHAT THIS PAPER ADDS:**

*What is already known on the subject*

- The existing literature is limited by varied and unclear methodological approaches to speech analysis and subtype classification, small sample sizes and a need for further comparison with a control group. Furthermore, no studies were found that assess speech in the mixed subtype.

*What this paper adds to the existing knowledge*

- In diadochokinesis and monologue, the nontremor‐dominant subtype showed shorter production time and, consequently, a smaller number of syllables when compared to the control group. The mixed subtype did not differ from the control group, performing similarly in all tasks. Significant differences were between the mixed subtypes and nontremor‐dominant regarding the average duration of the syllables in the monologue task.

*What are the potential or actual clinical implications of this work?*

- This study has a limitation: assessments were performed during the ON phase of medication, which may have masked some speech changes, especially in the dominant tremor subtype. These findings represent a pattern observed across subtypes at a single time point in a cross‐sectional study. As such, they may not reflect changes or developments that may occur over time or in different contexts. Therefore, as part of future perspectives, it is not yet known whether these results persist over a long period. We encourage researchers to conduct longitudinal studies with this population to better identify speech changes.

## 1 Introduction

Parkinson's disease (PD) is a progressive neurodegenerative disorder primarily associated with the characterized by the loss of dopaminergic neurons in the substantia nigra (Braak et al. 2003; Poewe et al. 2017; Simon et al. 2020). It is considered the most common type of Parkinsonism, more frequently observed in individuals over 50 years of age and in males, with prevalence increasing with advancing age (GBD 2016 Parkinson's Disease Collaborators 2018). The motor symptoms typically include bradykinesia with either rest tremor, rigidity or both, and nonmotor symptoms (Postuma et al. 2015). Additionally, PD is often associated with secondary symptoms such as loss of smell, sleep dysfunction, autonomic dysfunction, psychiatric disorders, cognitive impairment, as well as speech and swallowing difficulties (Armstrong and Okun 2020; Chaudhuri and Sauerbier 2016; Morris et al. 2019. Given its chronicity and progression nature, PD poses a considerable economic and public health burden (Ou et al. 2021).

PD is generally considered to have several motor subtypes (Adams et al. 2023). The classification of these subtypes is often based on disease features. It is commonly assessed according to the Movement Disorders Society – Unified Parkinson's Disease Rating Scale (MDS‐UPDRS), which includes motor and nonmotor sections and can be utilized to help define subtypes. However, it does not establish subtype classifications in itself. Based on clinical assessments, PD subtypes are frequently categorized into tremor‐dominant, nontremor‐dominant and mixed/indeterminate groups (Goetz et al. 2008; Jankovic 2008; Adams et al. 2023; Marras et al. 2024). Recently, the MDS task force has discussed the limitations of this classification and proposed recommendations to improve the definition and characterization of PD subtypes (Mestre et al. 2021; Tosin et al. 2020). Among the key recommendations was the inclusion of both motor and nonmotor symptoms to enhance the comprehensiveness and accuracy of subtype classifications (Luo et al. 2019). Although the MDS‐UPDRS was not specifically designed for subtype classification, studies have proposed approaches to categorize PD subtypes based on specific items from the scale. Goetz et al. (2008) highlight that although the MDS‐UPDRS does not define subtypes, it can be combined with specific item selections to differentiate between them. Tosin et al. (2020) utilized an Item Response Theory (IRT) analysis to examine the motor examination section of the MDS‐UPDRS and found that tremor and nontremor items could distinguish between PD motor subtypes. Similarly, Lou et al. (2019) analysed data from the BioFIND study and suggested that motor phenotype classification in moderate to advanced PD can be derived from specific MDS‐UPDRS items. These findings align with a theoretical framework supporting the possibility of identifying PD subtypes based on a selection of motor and nonmotor items. This perspective suggests that while the MDS‐UPDRS does not provide an explicit classification system, it remains a valuable tool for advancing our understanding of PD heterogeneity.

PD subtypes have been associated with different clinical characteristics, genetic patterns and the likelihood of developing complications. The tremor‐dominant phenotype has been suggested to exhibit a relatively slower progression and may be more frequently characterized by resting tremor and abnormal gait (Prime et al. 2020; Josephs et al. 2006; Gong et al. 2023). Conversely, the nontremor‐dominant phenotype has been linked to a more rapid progression and more significant motor deficits, including more pronounced axial motor symptoms, bradykinesia and rigidity (Zetusky et al. 1985; Prime et al. 2020; Zuo et al. 2017). Some studies indicate that individuals with the nontremor‐dominant subtype may experience a higher prevalence of swallowing impairment and speech changes (Thijs and Dumican 2023), along with a greater likelihood of cognitive decline compared to those with the tremor‐dominant subtype (Gong et al. 2023). Furthermore, research suggests that the risk of developing dementia and neuropsychiatric disorders may be higher in individuals with nontremor‐dominant and mixed subtypes compared to those with the tremor‐dominant subtypes (Burn et al. 2006; Alves et al. 2006; Jankovic et al. 1990; van der Hoek et al. 2011; Berg et al. 2021). The authors emphasize the need for longitudinal and multimodal studies to further investigate these subtypes, particularly in the prodromal phase, to improve disease understanding and facilitate more targeted and effective intervention strategies (Berg et al. 2021, 2015).

There is growing interest in whether motor subtypes may be associated with distinct patterns of speech disruption patterns of speech disruption (Goberman 2005; Skodda and Schlegel 2008). Some studies have reported differences in phonation between tremor‐dominant and nontremor‐dominant subtypes (Burk and Watts 2019), while others, such as Brown and Spencer (2020), found no significant differences in phonation variables, prosody or fluency between these groups. Additionally, research by Rusz et al. (2023) suggests that individuals classified within the tremor‐dominant group may exhibit less severe speech impairment and a more favourable prognosis regarding speech disorders. However, these individuals may still experience a decline in vocal quality, articulatory precision, timing abnormalities and monopitch.

It remains unclear to what extent the underlying mechanisms of different PD subtypes influence speech impairment. Further research is essential to understand these relationships better, as this knowledge may facilitate earlier intervention, support individualized care and optimize healthcare resources (Johansson et al. 2023). Identifying speech characteristics associated with PD subtypes could also contribute to the development of biomarkers in future studies. However, existing literature on this topic is constrained by variability in speech analysis methodologies, inconsistent subtype classifications, small sample sizes and limited comparisons with control groups (CGs). These challenges are particularly evident when considering the mixed subtype (Brown and Spencer 2020; Burk and Watts 2019; Park et al. 2014). Given these limitations, the present study aimed to (1) compare the auditory‐perceptual assessment of speech across PD motor subtypes and (2) evaluate the acoustic characteristics of speech in comparison to a CG.

## 2 Material and Methods

### 2.1 Participants

The study included 115 individuals diagnosed with PD recruited from the Neurology Service (Movement Disorders Outpatient Clinic and Speech Therapy Outpatient Clinic) of a university hospital in Brazil. Data collection was between June 2021 and June 2022. Eligibility criteria included a clinical diagnosis of idiopathic PD confirmed by a neurologist and Brazilian Portuguese as the native language.

A control group comprising 15 healthy participants was recruited from the community. Exclusion criteria for both groups included a history of prior neurological events, motor disorders, systemic diseases or structural alterations affecting voice and/or speech, as well as severe hearing loss and/or a dementia diagnosis.

Clinical and sociodemographic data were obtained during the medical consultation and/or extracted from electronic medical records, while data for the CG were collected during the evaluation session. The study was approved by the Local Research Ethics Committee (protocol number 2019‐0789), and informed consent was obtained from all participants

### 2.2 Motor Subtype Classification

Subtype classification followed the MDS‐UPDRS guidelines, where higher scores indicate greater impairment of PD symptoms. The classification was based on the tremor‐dominant to postural instability and gait difficulty ratio (nontremor dominant ‐ PIGD) as follows: tremor‐dominant (TD) subtype ratio ≥ 1.5; nontremor‐dominant (PIGD) subtype ratio ≤ 1.0 and mixed subtype ratio > 1.0 but < 1.5 (Adams et al. 2023; Jankovic et al. 1990; Stebbins et al. 2013). Using this classification, 61 individuals with PD (53%) were categorize as tremor‐dominant, 39 (34%) as nontremor‐dominant and 15 (13%) as the mixed subtype. A neurologist assessed and assigned the MDS‐UPDRS scores. Our data indicate that 62.6% of the sample had an onset before 50 years, with the following distribution: tremor‐dominant (67.21%), nontremor‐dominant (56.41%) and mixed (60%).

### 2.3 Data Collection

**FIGURE 1:**
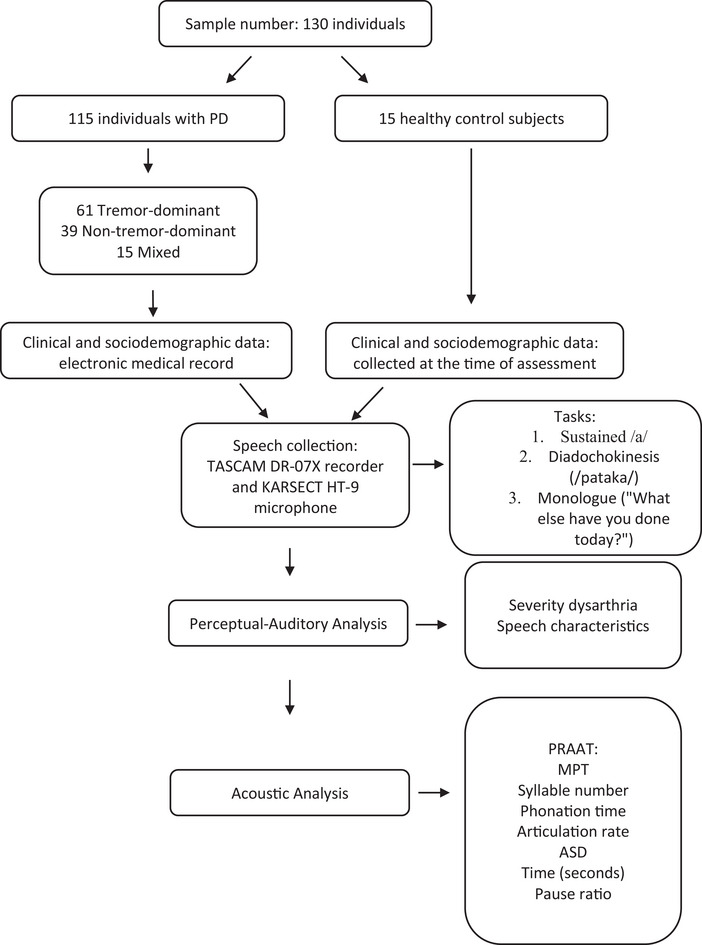
Data collection flowchart. ASD, average syllable duration; MPT, maximum phonation time (sustained vowel).

Figure 1 presents a flowchart summarizing the data collection process.

### 2.4 Speech Recording Procedure

Speech samples were recorded in a quiet environment using a portable TASCAM DR‐07X recorder and a KARSECT HT‐9 headset microphone. The microphone was positioned approximately 5 cm from the speaker's mouth, outside the main exhalation stream, ensuring a fixed distance and avoiding air currents from speech production. A unidirectional microphone with phantom technology was used, minimizing electromagnetic interference since power is delivered through the cable. A single trained researcher conducted all speech samples during 30‐min sessions. All individuals were on dopaminergic medication at the time of recording. The recordings were sampled at 44.1 kHz, quantized at 16 bits (Rusz et al. 2021) and later transferred to a computer for analysis using the PRAAT software (version 6.1.55) as commonly done in clinical settings outside dedicated speech labs.

### 2.5 Speech Tasks and Evaluation Sequence

Each participant completed the following speech tasks in a standardized sequence:

1. Sustained /a/ – participants produced a sustained vowel /a/ in a single breath for as long as possible;
2. Diadochokinesis (DDK) – participants performed “sequential movement rates” by rapidly repeating /pataka/ in a single expiration (Pierce et al. 2013);
3. Monologue – participants spoke for 60 s, responding to the question: “What have you done today since waking up?”

Before analysis, all recordings underwent a preliminary quality check to ensure they met the required acoustic criteria.

### 2.6 Auditory Perceptual Analysis

**FIGURE 2:**
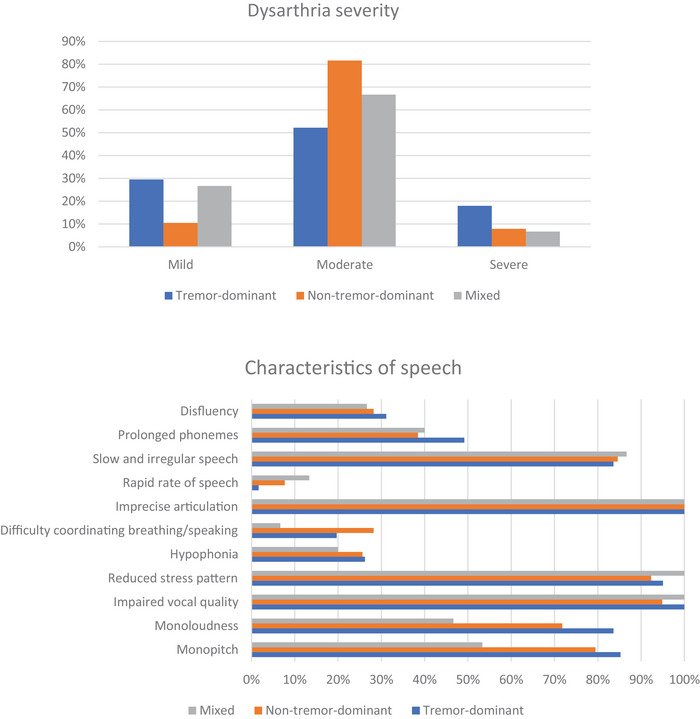
Speech characteristics and degree of dysarthria through auditory‐perceptual analysis.

The auditory‐perceptual analysis assessed speech characteristics and dysarthria severity using sustained /a/ vowel, diadochokinesis and monologue tasks. Three trained speech therapists, each with at least 7 years of experience and blinded to the participant's diagnosis, evaluated the audio recording in randomized order. Before analysis, the professionals participated in a brief auditory training session to ensure consistency in assessment. The raters were not trained using the actual data samples from the participants in the study to avoid potential bias. Instead, they were trained using separate audio recordings from individuals with varying dysarthria severities that were not part of the study dataset. The training session involved listening to these reference samples, discussing key speech features and aligning perceptual criteria to ensure consistency across raters. This process aimed to enhance reliability while minimizing potential bias in the ratings. The speech therapist was asked to choose between the following options when listening to the tasks: monopitch, monoloudness, impaired vocal quality, reduced stress pattern, hypophonia, difficulty in coordinating breathing and speech, imprecise articulation, fast speech rate, slow speech, prolonged speech phonemes and dysfluency (Dashtipour et al. 2018; Muñoz‐Vigueras et al. 2021; Di Pietro et al. 2022). Each audio was rated on six dimensions (overall dysarthria severity as well as respiration, resonance, phonation, prosody and articulation), each via a four‐point scale (0 = normal, 1 = mildly impaired, 2 = moderately impaired, 3 = severely impaired) (Duffy 2019). For the classification of dysarthria severity, excellent agreement was obtained between the evaluators (Kappa Index coefficient ≥ 0.90). The evaluators listened to the complete audio recordings, classified the motor bases and speech characteristics and then provided an overall severity score of the dysarthria from 0 to 3. Each evaluator independently assigned the final dysarthria grade, and in case of disagreement, another independent evaluator analysed the audio. Figure 2 shows the percentage of dysarthria among the motor subtypes of the disease.

### 2.7 Acoustic Speech Analysis

The sound files were manually marked to determine the beginning and end of vowel production, with the durations automatically extracted for each participant using a custom script. This script also automatically detected intensity peaks for the DDK and monologue tasks.

In Brazilian Portuguese, vowels occur only in the syllable nucleus, so counting the intensity peaks is equivalent to determining the number of syllables produced. The reliability of this automated method was validated by comparison to manual acoustic analysis (de Jong and Wempe 2009). The parameters used in the analysis were adapted from (Rusz et al. 2011) and (Vogel and Maruff 2008). For participants who spoke for less than 60 s, pauses were included as a variable in the analysis, with no temporal truncation of the speech signal based on time.

The following variables were considered for the articulation tasks: number of syllables, duration (seconds), phonation time (seconds), articulation rate (−), average syllable duration – ASD (seconds) and pause ratio (percentage).

The pause ratio variable was used only for the monologue task. This variable measures the pause time between articulated words, capturing moments when the participant is not speaking.

For the DDK task, participants are instructed to repeat the sequence as quickly as possible in a single breath. In this task, phonation time was measured based on the total duration of the task, rather than individual syllables, since there is usually no pause between syllables. The total duration of the sequence corresponds to the phonation time. It is important to note that in this study, a pause is defined as the silent interval in the audio signal – that is, the absence of sound. Thus, the pause ratio in the monologue represents the proportion of time the participant is not speaking. Furthermore, as the task requires language production, the pause may indicate breathing moments, hesitation while deciding what to say next or a prosodic grouping of speech sounds.

### 2.8 Statistical Analysis

The independent variables (gender, age, education, disease duration, age of symptom onset, HY, UPDRS, intelligibility and degree of dysarthria) were presented as descriptive analyses (absolute and relative frequencies, mean and standard deviation, median and interquartile range). Statistical tests were selected based on the Kolmogorov–Smirnov and Shapiro–Wilk tests. The nonparametric Kruskal–Wallis test was used to compare speech variables between independent groups. The chi‐square test was used to compare speech characteristics between groups through perceptual analysis. Statistical significance was defined as *p* < 0.05. The statistical software used was SPSS version 22.0.

## 3 Results

Table 1 presents a comparison of sociodemographic characteristics across the four groups: tremor‐dominant, nontremor‐dominant, mixed and CG. No statistically significant differences were observed among the groups regarding gender, age or education level. The tremor‐dominant subtype demonstrated a later onset age of symptoms and a higher UPDRS score. However, no statistically significant differences were observed between the subtypes regarding clinical data. The nontremor‐dominant group tended to have longer disease duration and higher HY scores.

**TABLE 1:** Sociodemographic data.

	Tremor‐dominant	Nontremor‐dominant	Mixed	Control group	*p* value
	(*n* = 61)	(*n* = 39)	(*n* = 15)	(*n* = 15)	
Male	39 (63.9%)	20 (51.3%)	9 (60%)	7 (±46.7%)	0.492
Age (years)	61 (53–66)	63 (56.7–70.5)	60 (55–63)	68 (61–72)	0.093
Education (years)	8 (5–12)	8 (5–12)	8 (5–18)	5 (4–16)	0.539
Disease duration (years)	12 (7.5–17)	15 (10.5–21)	12 (7–20)	–	0.069
Age of onset (years)	47 (40–54)	47 (37–54)	47 (35–51)	–	0.671
HY	2 (1–3)	3 (1–4)	1 (1–3,25)	–	0.240
MDS‐UPDRS (Part III)	12 (5–22)	12 (2–21)	7 (1–15)	–	0.433

*Note*: The values in the table are represented as: median (interquartile range). Except for sex, which is represented as a percentage.

Abbreviations: HY, Hoehn and Yahr Disability Stage Scale; MDS‐UPDRS, Movement Disorders Society – Unified Parkinson's Disease Rating Scale.

Figure 2 illustrates the classification of dysarthria severity among disease subtypes, which did not reach statistical significance (Chi^2^ = 8.15, d.f. = 4, *p* = 0.086). Additionally, no significant differences were found between the groups regarding speech characterization. Nevertheless, articulatory imprecision was commonly observed across subtypes and was present in all individuals.

**FIGURE 3:**
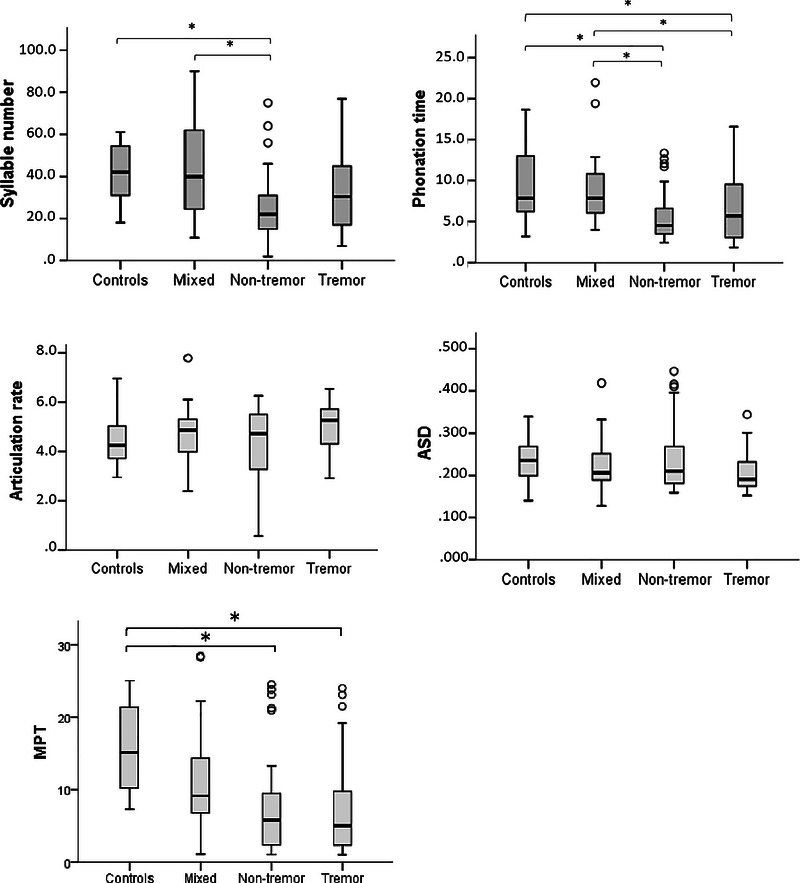
Group comparisons of acoustic variables for the diadochokinesis and sustained vowel MPT task. ASD, average syllable duration; MPT, maximum phonation time (sustained vowel); *p < 0.05. Measures: number of syllables (−), phonation time (seconds), articulation rate (−), ASD (seconds) and MPT (seconds).

Figure 3 shows a comparison of the acoustic articulation variables of the groups evaluated through the DDK and sustained vowel MPT tasks. In diadochokinesis, individuals with the nondominant tremor subtype differed from the following groups: controls [number of syllables (*p* = 0.027) and phonation time (*p* = 0.009)]; mixed [number of syllables (*p* = 0.030) and phonation time (*p* = 0.035)]. Thus, it can be observed that the motor subtype group presented shorter phonation time and lower syllable production compared to the control and mixed groups. In contrast, the dominant tremor subtype presented shorter phonation time than the control (*p* = 0.028) and mixed (0.012) groups, but no significant differences were observed in the number of syllables produced.

Figure 3 also shows the comparison between the groups regarding the maximum phonation time of sustained voice. The following median values (interquartile range) were observed: tremor‐dominant 5.04 (2.32–9.82), nontremor‐dominant 5.78 (2.34–9.74), mixed 9.11 (5.66–16.18) and controls 15.12 (10.14–22.77). Statistical analysis revealed a significant difference between the groups (*p* < 0.001). Post hoc comparisons indicated significant differences between the nontremor‐dominant and CGs (*p* < 0.001) and tremor and controls (*p* < 0.001).

**FIGURE 4:**
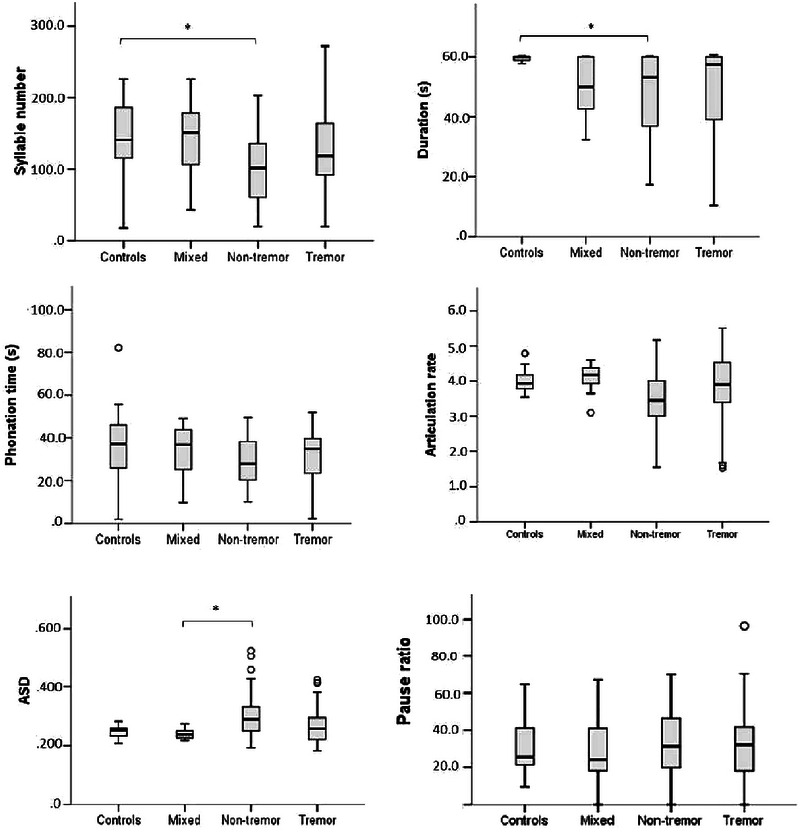
Comparisons by groups of the acoustic variables for the monologue task. ASD, average syllable duration; *p < 0.05. Measures: number of syllables (−), duration (seconds), phonation time (seconds), articulation rate (−), average syllable duration – ASD (seconds) and pause ratio (percentage).

Figure 4 compares the groups based on acoustic variables in the monologue task. The following differences were observed between the nontremor dominant group and controls: number of syllables (*p* = 0.026) and duration (*p* = 0.003). Thus, individuals with a nontremor dominant subtype demonstrated shorter task duration and consequently produced fewer syllables than the CG. In addition, these individuals with a nontremor dominant subtype also exhibited a slower average syllable production (*p* = 0.045) compared to the mixed subtype.

## 4 Discussion

This study aimed to explore whether speech characteristics differ among PD motor subtypes and the CG. The findings suggest significant differences in the maximum phonation time between the CG and both tremor‐dominant and nontremor‐dominant subtypes. Additionally, in diadochokinesis and monologue tasks, the nontremor‐dominant subtype appeared to exhibit shorter production time and fewer syllables compared to the CG. In contrast, the mixed subtype did not show significant differences from the CG, suggesting similar performance across all tasks.

These findings may provide further insights into the role of articulation changes in communication functionality among PD motor subtypes. This study also sought to address gaps in the literature, such as those reported by Brown and Spencer (2020), where no differences were observed between tremor‐dominant and nontremor‐dominant subtypes. Some methodological limitations in prior studies, including sample size, the inclusion of predominantly mild dysarthria cases and the absence of a CG, may have influenced those findings. By addressing these factors, the present study identified distinct speech among PD subtypes compared to the CG.

One of the key findings of this study is that the nontremor‐dominant subtype appeared to perform more poorly in articulation tasks compared to other subtypes and the CG. This observation is consistent with prior suggestions that dysarthria in PD may be associated with axial motor impairment, similar to postural instability and gait disturbances (Moustafa et al. 2016). Given that the nontremor‐dominant subtype has been linked to more rapid and severe disease progression, along with greater deterioration in both motor and nonmotor domains, these factors may contribute to more pronounced speech impairment in this group (Dumican and Watts 2020). Additionally, previous research has indicated that the tremor‐dominant subtype tends to show a better response to dopaminergic medications compared to the nontremor‐dominant subtype, which may also help explain some of the observed differences (Moustafa et al. 2016).

Another notable observation is that individuals with the nontremor‐dominant subtype tended to exhibit shorter production time and a lower number of syllables in diadochokinesis and monologue tasks compared to the CG. These findings are in line with previous studies reporting similar speech patterns in this population (Rusz et al. 2023; Tykalová et al. 2020). Additionally, prior research has identified a correlation between the UPDRS bradykinesia scores and voice measures in individuals with the nontremor‐dominant subtype (Suphinnapong et al. 2021), suggesting that bradykinesia may contribute to reduced phonation time and other speech alterations, as has been frequently observed in this subtype (Zetusky et al. 1985; Galaz et al. 2018; Cavallieri et al. 2023).

In addition to these findings, individuals with the nontremor‐dominant subtype appeared to have longer average syllable duration during spontaneous speech. This observation may be related to the characteristic phenotype of this subtype. Rusz et al. (2023) reported a correlation between reduced voicing onset time in a rapid syllable production task and decreased step count, irregular movement and prolonged pauses during walking, suggesting a possible association between speech and gait patterns in the nontremor‐dominant subtype. Additionally, Rusz (2023) found that vocal alterations were more prevalent in severe dysarthria, which may align with our findings. Given that the nontremor‐dominant sample in our study included individuals with more pronounced motor and speech impairments, this could partly explain the observed speech characteristics. Previous research has suggested that speech phenotypes may have the potential to predict the response to levodopa therapy in de novo PD (Rusz et al. 2021); In this study, three distinct speech subtypes in treatment‐naive PD patients: prosodic, phonatory‐prosodic and articulatory‐prosodic. Notably, the articulatory subtype is associated with more severe axial gait and cognitive impairments, which may correspond to more significant cortical atrophy linked to articulatory deficits. Additionally, digital speech biomarkers have been proposed as a useful tool for capturing disease progression and treatment effects, with potential applications in differential diagnosis (Rusz et al. 2024).

Interestingly, no significant differences were observed between the mixed subtype and the CG. This finding suggests that individuals classified within this group may have performed similarly to healthy controls across the examined speech task. One possible explanation for this could be that some individuals in this group had not yet transitioned to another motor subtype. Alves et al. (2006) found that over 8 years, 66% of patients initially classified as indeterminate subtype progressed to the nontremor‐dominant subtype, while 16% transitioned to the tremor‐dominant subtype, with only a small portion maintaining a mixed subtype classification. An alternative explanation for the absence of significant differences may be the smaller sample size of this group, which could have limited the ability to detect changes. Given the limited research on speech characteristics in the mixed subtype, further studies are warranted to understand this population better.

The speech tasks employed in this study were chosen to capture distinct aspects of speech production. Although the monologue task allows for the observation of linguistic and cognitive organization, maximum performance tasks primarily assess motor speech control. This dual approach aimed to account for both cognitive and motor influences on speech production. However, it is important to acknowledge that the cognitive demands of the monologue task may have influenced performance beyond motor speech impairment alone. The number of syllables and the duration of the monologue may provide additional insights, as these measures could also reflect underlying cognitive difficulties. Previous studies (Dos Santos et al. 2023) comparing different neurodegenerative conditions, such as PD and spinocerebellar ataxia Type 3 (SCA3), have indicated that some individuals with PD do not complete a 60‐s monologue task, which may suggest cognitive or linguistic challenges rather than purely motor‐related adjustments.

Regarding auditory‐perceptual assessments, no significant differences were observed between the PD subtypes in terms of dysarthria severity or speech characteristics. However, this method remains a crucial component of clinical evaluation, particularly in the early stages of speech impairment. Since the present study included relatively young participants, the perceptual differences may have been subtle. Nevertheless, auditory‐perceptual analysis remains the gold standard for diagnosing speech disorders (Duffy 2019). When combined with acoustic analysis, this approach may facilitate a more comprehensive understanding of speech alterations and support in clinical decision‐making. The integration of both methodologies could enhance the early detection of subtypes’ specific speech differences, contributing to the classification of nonmotor symptoms.

The Movement Disorder Society Task Force defines early‐onset Parkinson's disease (EOPD) with an upper cutoff of 50 years (Mehanna et al. 2022). Our study included a high proportion of individuals with EOPD, defined as an age of onset below 50 years, comprising 62.6% of the sample. The distribution of EOPD cases across motor subtypes was as follows: tremor‐dominant (67.21%), nontremor‐dominant (56.41%) and mixed (60%). Although EOPD is often considered a distinct subgroup due to differences in disease progression and nonmotor symptoms, studies, including those by Sharpe et al. (2020), suggest that its impact on speech production is not fundamentally different from later‐onset PD. Early‐stage PD, including EOPD, is characterized by subtle impairments in voice quality, articulation precision and speech timing, as shown in studies like Rusz et al. (2011). Despite these distinctions, the effects on speech and motor symptoms between early‐ and late‐onset PD show significant overlap, indicating that similar therapeutic approaches may be effective for both groups. However, EOPD is associated with a higher prevalence of rigidity, which aligns with our findings in the nontremor‐dominant group Postuma et al. 2015.

Overall, these findings highlight the need for further studies investigating speech characteristics across PD subtypes. A deeper understanding of these differences may aid in the identification of speech‐based biomarkers for disease monitoring and personalized therapeutic strategies.

### 4.1 Limitations and Future Directions

This study has certain limitations. Assessments were conducted during the ON phase of medication, which may have influenced the observed speech changes, potentially reducing the visibility of some speech changes, particularly in individuals with the tremor‐dominant subtype. These findings represent patterns identified at a single time point within a cross‐sectional design and may not fully capture variations that could emerge over time or in different conditions. Consequently, it remains uncertain whether these results persist longitudinally. Given the potential influence of disease onset on motor symptoms, future research – particularly longitudinal studies – should further explore whether speech impairments differ between early‐ and late‐onset PD and investigate speech changes in this population over time.

## 5 Conclusions

Differences in speech characteristics were observed among PD motor subtypes. The nontremor‐dominant subtype appeared to exhibit poorer speech performance compared to other subtypes and the CG across the sustained vowel, diadochokinesis and monologue tasks. Tremor‐dominant symptoms were primarily associated with phonation‐related changes, while nontremor‐dominant symptoms appeared to have a greater impact on articulation. Although the mixed subtype also exhibited speech alterations, its speech patterns were more comparable to those of the CG than to those of the other PD subtypes.

## Conflicts of Interest

The authors declare no conflicts of interest.

## Data Availability

All data generated or analysed during this study are included in this article. Further inquiries can be directed to the corresponding author.
